# Effect of the Number of Micro-Osteoperforations on the Rate of Tooth Movement and Periodontal Response in Mice

**DOI:** 10.3389/fphys.2022.837094

**Published:** 2022-03-03

**Authors:** Tselmuun Erdenebat, Dong-Joon Lee, Su-Jung Kim, Yoon Jeong Choi, Eun-Jung Kim, Eun-Hack Choi, Jing Liu, Chung-Ju Hwang, Han-Sung Jung, Jung-Yul Cha

**Affiliations:** ^1^Department of Orthodontics, The Institute of Craniofacial Deformity, Yonsei University College of Dentistry, Seoul, South Korea; ^2^Division in Anatomy and Developmental Biology, Department of Oral Biology, Taste Research Center, BK21 FOUR Project, Yonsei University College of Dentistry, Seoul, South Korea; ^3^Department of Orthodontics, Kyung Hee University School of Dentistry, Seoul, South Korea

**Keywords:** micro-osteoperforation, orthodontic tooth movement, micro-CT, root resorption, cementum, bone formation

## Abstract

Accelerated tooth movement can be achieved using micro-osteoperforations (MOPs) to stimulate regeneration of the alveolar bone during minimally invasive surgical trauma. However, there is currently no standardized protocol and limited reports regarding the side effects of MOPs based on biological evidence. This study sought to evaluate the biological effects of the number of MOPs on orthodontic tooth movement (OTM) and the potential risk for root resorption. Male CD1 mice were divided into 4 groups based on the number of MOPs, as follows: Sham; 0MOP+OTM; 2MOP+OTM; and 4MOP+OTM groups. Tooth movement distance and the number of osteoclasts were higher whereas bone volume and trabecular number were lower in the 4MOP+OTM group compared to those of the 0MOP+OTM group. Immunofluorescent assay analysis indicated that the 4MOP+OTM group was positively associated with rapid cementum regeneration and periodontal ligament tissue formation. Our findings revealed that the MOP procedure affected tooth movement and did not significantly contribute to root resorption, whereas it may promote constitutive activation of cementogenesis.

## Introduction

In orthodontic treatment, one of the main objectives is to reduce the duration of the orthodontic treatment without contributing to side effects such as root resorption. Orthodontic tooth movement (OTM) uses a mechanical force to induce tooth movement, but it may also couple bone resorption and bone formation (Krishnan and Davidovitch, 2006). To modulate these biological processes, various surgical interventions have been introduced to accelerate tooth movement based on the regional acceleratory phenomenon (RAP) (Murphy et al., 2014; Tsai et al., 2016; Zou et al., 2019).

The RAP, first introduced by Frost (1983), is biological reaction that described the localized self-demineralization process in the alveolar bone. RAP is characterized at the cellular level by increased activation of the basic multinuclear units (BMUs), thereby increasing the bone remodeling rate. RAP occurs typically in the periodontal tissue after surgical operation and affects the rate of tooth movement under orthodontic force (Verna, 2016).

The surgical interventions were developed in combination with different levels of invasive techniques including traditional surgical procedures such as corticotomies (Kole, 1959; Wilcko et al., 2009), and distraction of alveolar bone (Uzuner and Darendeliler, 2013). Despite augmenting accelerated tooth movement, these methods have had limited clinical use due to their invasiveness and local side effects, such as bone loss, osteoporosis, and delayed wound healing (Long et al., 2013; Kalemaj et al., 2015). For these reasons, minimally invasive methods were introduced including piezocision (Dibart et al., 2009), corticision (Kim et al., 2009), and micro-osteoperforations (MOPs) (Teixeira et al., 2010). These methods are more widely used in clinical practice, because patients experience less pain and there is high acceptance of the surgical procedure. Nevertheless, the clinical efficacy of minimally invasive surgical methods was not clearly confirmed by clinical studies because there are contradictory results from different procedures (Mheissen et al., 2020; Sivarajan et al., 2020).

Among the minimally invasive methods recently developed, MOP is being applied clinically by creating shallow perforations in the alveolar bone adjacent to the target tooth without the need for bone grafting or flap surgery (Alikhani et al., 2015). According to clinical reports evaluating the rate of canine retraction over the short term (1–6 months), MOPs accelerated tooth movement 2–3-fold (Alikhani et al., 2013; Agrawal et al., 2019; Kundi et al., 2020). MOPs were reported to increase the expression of inflammatory markers, as well as the rate of tooth movement (Al-Khalifa and Baeshen, 2021) in rat models. Due to the fundamental characteristics of MOPs, this minimally invasive surgical intervention has reported benefits regarding treatment time in both animal and clinical research, but exhibited varying results concerning root resorption as a side effect of MOP (Shahabee et al., 2020). Root resorption is a common physiological resorptive and reparative process that may be triggered by mechanical stimuli and inflammation (Henry and Weinmann, 1951). An animal study demonstrated that 8.9% of total root volume decreased with MOP application compared with a control group (Chen et al., 2020). Another clinical study reported that the total average volumetric root loss of premolars treated with MOPs was 42% greater than that of the traditional orthodontic treatment group (Chan et al., 2018). There are limited reports and the relationship between micro trauma and root resorption remains unclear. In addition, a study investigating the physiological mechanism of root repair is required because cementum remodeling is a continuous process during orthodontic treatment (Turkkahraman et al., 2020). Moreover, MOP methods differ in terms of the number of holes, sizes, and applied force, as well as whether with a flap or flapless.

Recently, it was reported that Wnt-responsive (Axin2) tissue, and cells were part of the periodontal ligament (PDL) that participated in alveolar bone healing (Yuan et al., 2018) and PDL cells play a fundamental role in root formation (Lohi et al., 2010). Wnt/β-catenin PDL cells induced cementoblast differentiation by triggering Osx expression and regulated cementogenesis and cementum mineralization (Choi et al., 2017; Xie et al., 2019). Dentin matrix protein 1 (DMP1) and bone sialoprotein (BSP) may have similar functions as early osteoblast markers, and they are expressed in mineralized specific tissues, such as in dentin formation and cementum mineralization (Feng et al., 2003; Baloul et al., 2011). In addition, DMP1 was recommended as a biomarker for cementocytes and in the developing cementum mass (Choi et al., 2016).

To investigate the rate of tooth movement and periodontal tissue damage arising from MOP, this study evaluated the rate of tooth movement and the risk for root resorption and remodeling of the periodontium resulting from different numbers of MOP combined with an applied orthodontic force in a mouse model. We observed how a different number of MOPs affects the rate of tooth movement and the risk of root resorption, as well as the quality and quantity of alveolar bone. We also investigated whether the Wnt signaling pathway including Axin2, β-catenin, BSP, and DMP1 plays a critical role in the regenerative process of the root surface and PDL space due to the MOP procedure.

## Materials and Methods

All animal sections, preparations, and surgical protocols were conducted according to the Association for Assessment and Accreditation of Laboratory Animal Care (AAALAC) international guidelines and approved by the Yonsei University Health System - Institutional Animal Care and Use Committee (YUHS-IACUC) (Approval No. 2018-0052).

### Animals

Thirty-six heads of 8-week-old male CD1 mice weighing 35–40 g were used in this study. 12 mice were randomly divided into three experimental group (0MOP+OTM only group, 2MOP+OTM group, and 4MOP+OTM group), and the 12 collateral parts (non-OTM) of the experimental site were randomly selected from 4 mice of each experimental group, which comprised the Sham group. All animals were housed in a temperature-controlled room (22°C) under artificial illumination with a 12-h light/dark cycle and 55% relative humidity. The mice were provided access to food and water ad libitum. All surgical procedures were performed under general anesthesia with ketamine-xylazine (0.10 mL/10 g).

### Force and Micro-Osteoperforation Application

A 0.009-inch stainless steel ligature (GAC international, Bohemia, NY) was placed around the contact between the first and second right maxillary molars and securely ligated. Another 0.009-inch stainless steel ligature was then ligated to the maxillary incisors, and orthodontic force was applied using a super-elastic nickel titanium (NiTi) closed-coil spring with a 25 g force (EW, JISCOP, Korea) and attached to these ligatures (Supplementary Figure 1A). After a self-etching primer and light cure adhesive composite resin (Transbond Plus; 3 M Unitek, Monrovia, California, United States) were applied to the maxillary incisors to prevent slippage of the ligature wire. Mice in the MOP group received two and four shallow perforations on the palatal alveolar bone around the maxillary first molar, after which drilling was performed and fully immersed into the bone for every perforation (Supplementary Figures 1B–E). These perforations were created using a ¼ round bur (Komet, Germany) with a low-speed hand piece. Holes were created at a distance of 0.66 mm for two perforations, and 0.33 mm for four perforations. Subsequently, the mandibular incisors were trimmed, and a collar was worn to prevent appliance breakage. Two mice were kept per cage. A regular soft diet (Transgenic Dough Diet™, Product #S3472, BioServ) was provided during the experimental period. Signs of infection or prolonged inflammation was not observed. Body weight and appliance stability were checked daily.

### Measurement of Tooth Movement

OTM distance was measured under a stereoscope (Olympus Stereo zoom microscope SZ61, Tokyo, Japan). The images were obtained at a 0.67X and 1.5X magnification at a 2190X1640-pixel size (DIXI Imaging Solution, v.2.8). The following two parameters were used to measure tooth movement: (1) Tooth movement was calculated as the average of two reference lines, which corresponded to the central cusp and palatal groove of the maxillary first and second molars measured at the inter-proximal heights of the contour between the most mesial point of the second molar crown and the most distal point of the first molar crown (Supplementary Figure 2A). Finally, the mean value was calculated for the two reference lines. (2) The change in molar inclination, which is the angle of tooth inclination of the maxillary right (experimental) and left (Sham group) side was measured in a sagittal section of micro-CT image using a previously reported method (Verna et al., 2000) (Supplementary Figure 2B) with image-analysis software (ImageJ, ver. 1.38e, NIH, United States).

### Micro-CT Analysis of Surrounding Alveolar Bone and Root Resorption

**FIGURE 1:**
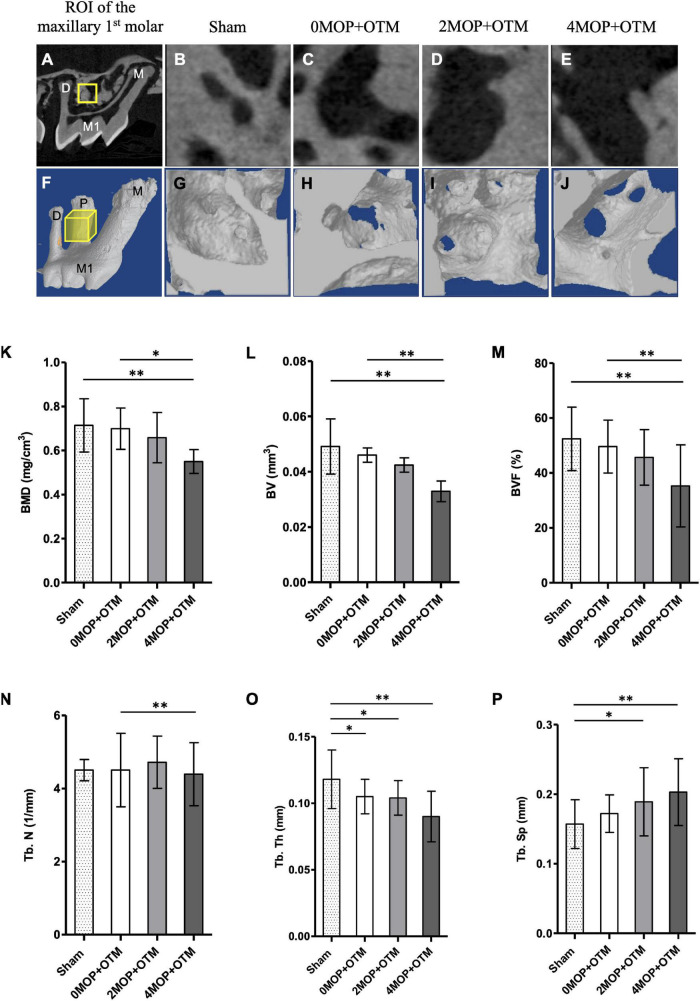
Micro-CT analysis for bone quality. **(A–E)** Sagittal section image of micro-CT. The ROI (yellow square on **A**) of alveolar bone quality for each group from **(B–E)**. **(F–J)** 3D reconstructed images of ROI (yellow cube on **F**) of experimental groups. According to the volumetric analyses, **(K)** BMD, mg/cm^3^; **(L)** BV, mm^3^; **(M)** BVF, % decreased in the 4MOP+OTM group (0.55 ± 0.18 mg/cm^3^; 0.03 ± 0.01 mm^3^; 35.27 ± 14.94 %; *p* < 0.01) compared to the 0MOP+OTM group (0.69 ± 0.09 mg/cm^3^; 0.04 ± 0.009 mm^3^; 49.59 ± 9.62 %) and Sham group (0.71 ± 0.12 mg/cm^3^; 0.04 ± 0.01 mm^3^; 52.39 ± 11.55 %). **(N)** Additionally, the Tb. N, 1/mm was found to decrease in the 4MOP+OTM group (3.78 ± 0.89 1/mm; *p* < 0.01) compared to the 0MOP+OTM group (4.71 ± 0.71 1/mm). **(O,P)** Regarding changes in the Tb. Th, mm; Tb. Sp, mm were different between the 4MOP+OTM group (0.09 ± 0.01 mm; 0.20 ± 0.04 mm; *p* < 0.01) and Sham group (0.11 ± 0.02 mm; 0.15 ± 0.03 mm). In addition, the 2MOP+OTM group (0.10 ± 0.01 mm; 0.18 ± 0.04 mm; *p* < 0.05) was different compared with the Sham group. (*N* = 12/each group) (M1, 1st molar; M, mesial; P, palatal; D, distal; data are expressed as mean ± standard deviation. Tested by Mann–Whitney *U*-test, at *p < 0.05; ^**^p < 0.01).

**FIGURE 2:**
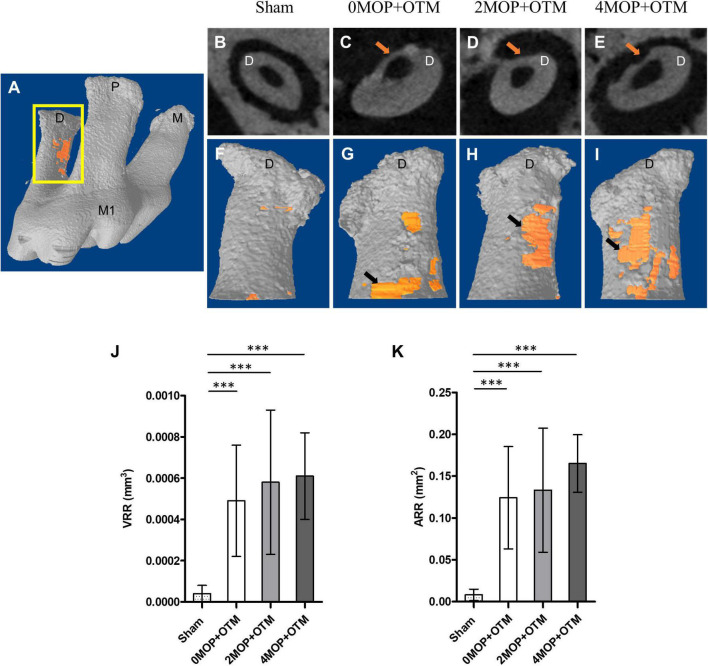
Micro-CT analysis for root resorption. **(A)** Maxillary right 1st molar, and the segment of the distal root that was analyzed for root resorption. View of the axial section at which the crater (*arrow*). **(F–I)** 3D reconstructed images of distal root mesial side with crater (*orange*) for each group based on **(B–E)**. **(J)** The volumetric analysis shows the VRR, mm^3^ in the 0MOP+OTM group averaged (0.00049 ± 0.00027 mm^3^), while those in the 2MOP+OTM group were (0.00058 ± 0.00035 mm^3^), and those in the 4MOP+OTM group averaged (0.00061 ± 0.00021 mm^3^). **(K)** In terms of measured ARR, mm^2^ that of the 0MOP+OTM group averaged (0.12 ± 0.06 mm^2^), the 2MOP+OTM group averaged (0.13 ± 0.07 mm^2^), and the 4MOP+OTM group (0.14 ± 0.05 mm^2^). These results were not significant between experimental groups. The Sham group’s value for VRR, mm^3^ (0.00004 ± 0.00004 mm^3^; *p* < 0.001), and ARR, mm^2^ (0.008 ± 0.007 mm^2^; *p* < 0.001) were decreased compared to those of the other groups. (*N* = 12 for each group) (M1, 1st molar; M, mesial; P, palatal; D, distal; arrow, indicate root resorption; data are expressed as means ± standard deviations. Tested by Mann–Whitney *U* test, at ^***^p < 0.001).

Mice were euthanized using a CO_2_ chamber, then the maxilla was dissected and fixed with neutral buffered formaldehyde (15%, pH 7.4; Duksan Pure Chemicals Co., Ltd., Ansan, Korea) solution for 24 h at 4°C. *In vivo* three-dimensional (3D) images were taken for each sample using high-resolution micro-CT (SkyScan 1173; BRUKER-MICROCT, Kartuizersweg 3B 2550 Kontich, Belgium) at a voltage of 90 kVp and electrical current of 88 μA with an X-ray source and a 5.33 μm pixel size. Serial transverse scan images were obtained at a resolution of 18 μm. Scanning data were reconstructed using NreconVer 1.6 (Recons v.1.6.10.4, Bruker). Three-dimensional images were reconstructed with the Data Viewer (DataViewer v.1.5.4.0 Skyscan, Bruker), CTvox (CT vox v.3.3.0, Bruker) program. The bone parameters and root resorption were analyzed by CTAn (CTAn v.1.17.7.1, Skyscan, Bruker). Bone volume and trabecular changes in the inter-radicular space of the maxillary first molar were assessed as quantitative analysis of alveolar bone changes. The ROI was selected to be cubic in shape (*450X450X450 μm*), and it was located 100 *μm* away from the mesial surface of distal root (Figure 1A square, Figure 1F cube). Volumetric quantification of the root was conducted following a previously established protocol (Crowther et al., 2017). The ROI (*450 μm*) was determined from the furcation to the border of acellular and cellular area (Figure 2A rectangular). Next, the threshold value for binary image was determined from 255 to 66 through all specimens, providing an accurate representation of the alveolar bone image within micro-CT images. Finally, the software calculated the changes in alveolar bone and volume of root resorption.

### Histological Analysis

After micro-CT images were obtained, the maxilla was hemisected and decalcified in ethylenediaminetetraacetic acid (EDTA; 15%, pH 7.4) for 6–7 weeks and then processed for standard paraffin embedding. Sagittal sections (4 μm thick) along the molars were obtained using a microtome (SP 1600, Leica DFC 290, Leica, Nussloch, Germany). Two serial sections from each animal were stained using hematoxylin and eosin (H&E), and three serial sections for tartrate-resistant acid phosphatase (TRAP), and immunofluorescence staining. The slides were deparaffinized with xylene and rehydrated before staining. TRAP staining was performed with a leukocyte acid phosphatase staining kit (Sigma Chemical, St Louis, Mo) according to the manufacturer’s instructions. Stained sections were scanned using microscope (Leica, DM 2500 LED, Wetzlar, Germany) at 20×/0.40 PH1 magnification. ROI (*0.27X0.42 μm*) was determined to be a rectangular box, including the cementum, periodontal ligament, and alveolar bone. TRAP-positive multinucleated cells in the compression were quantified as numbers and area of osteoclast with View Point Light (v1.0.0.9628) (Supplementary Figure 3).

### Immunofluorescence

After dehydration, the tissue sections were incubated with antigen retrieval solution proteinase K (10 μg/mL, AM2546, Thermo Fisher Scientific, United States) for 20 min at 37°C. The specimens were blocked with goat serum (0.1%) for 15 min at room temperature then incubated overnight with monoclonal rabbit anti-axin2 (Abcam; dilution 1:300), β-catenin (Santa Cruz Biotechnology, dilution 1:200), bone sialoprotein (BSP) (Abcam; dilution 1:200), dentin matrix protein 1 (DMP1) (LSBio, WA, United States; dilution 1:500) antibodies at 4°C. The specimens were incubated with goat anti-rabbit Alexa Flour 488 (Thermo Fisher Scientific, MA, United States; dilution 1:200) antibody and counterstained with DAPI (Molecular Probes, OR, United States; dilution 1:1,000). The sections were examined using a confocal laser microscope (DMi8; Leica, Wetzlar, Germany). The representative area for the expression, a square box of 100 μm, was placed on the compression and tension side of the alveolar bone, including the cementum, periodontal ligament, and alveolar bone. Immunostaining procedures were performed on at least three slides for each group of individual mice (biological replications), with consistent results.

### Statistical Analysis

All data for the groups were presented as means and standard deviations. The mean value was calculated and recorded as the final value. As a non-parametric alternative, the Kruskal–Wallis test with *post-hoc* test (Mann–Whitney U-test) were used to determine the statistical significance of the intergroup comparisons of tooth movement distance, bone parameters, root resorption and TRAP-positive cells. Spearman correlation analysis was conducted to analyze the correlation between the variables within the groups. For the results *, ^**^, and ^***^ describe *p*-values of < 0.05, 0.01, and 0.001, respectively. All statistical analyses were performed using SPSS software (version 25; IBM Co., Armonk, NY, United States).

## Results

### Tooth Movement by Orthodontic Force Increased With the Number of Micro-Osteoperforations

**FIGURE 3:**
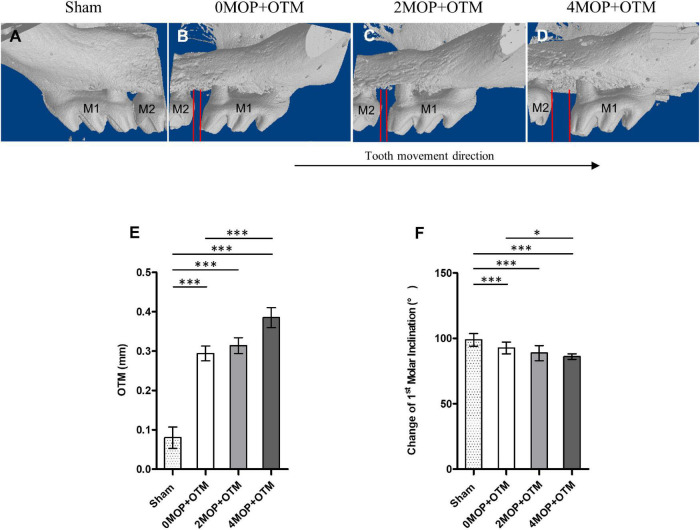
Comparison of the tooth movement distance and molar inclination. **(A–D)** 3D volumetric model (*buccal side*) of maxillary molar showing comparison of tooth movement distance between the groups after the experimental period. **(E)** The Sham group (0.08 ± 0.02 mm; *p* < 0.001) decreased the distance of OTM compared with the 0MOP+OTM (0.29 ± 0.06 mm), 2MOP+OTM (0.31 ± 0.07 mm) and 4MOP+OTM (0.38 ± 0.08 mm) groups. The 4MOP+OTM group showed increased OTM (*p* < 0.01) compared with the 0MOP+OTM group. **(F)** The Sham group exhibited increased 1st molar inclination (99.01 ± 4.91°; *p* < 0.001) compared with the 0MOP+OTM (90.52 ± 6.59°), 2MOP+OTM (88.74 ± 5.73°) and 4MOP+OTM (84.2 ± 7.42°) groups. The 4MOP+OTM group exhibited decreased OTM (*p* < 0.05) compared with the 0MOP+OTM group. (*N* = 12 for each group) (M1, 1st molar; M2, 2nd molar; red lines in **(B–D)** showed the tooth movement distance; data are expressed as mean ± standard deviation. Tested by Mann-Whitney *U*-test, *p < 0.05; ^***^p < 0.001).

A different number of MOPs were applied in CD1 mice with a continuous static force for mesial movement during 14 days (Figures 3A–D). In terms of distance of OTM (Figure 3E), the MOP groups were higher than the Sham group (0.08 ± 0.02 mm). The 4MOP+OTM group (0.38 ± 0.08 mm) exhibited significantly greater movement than the 0MOP+OTM group (0.29 ± 0.06 mm). Molar inclination (Figure 3F) in the 4MOP+OTM group was lower than that of the 0MOP+OTM group, whereas that of the 2MOP+OTM group showed no difference compared to the other groups. In addition, the Sham group exhibited a significant increase among the groups. Based on this result, molar distance, and inclination showed sharp increase in the 4MOP+OTM group. In contrast, the 2MOP+OTM group showed similar results to those of the 0MOP+OTM group.

### Quality and Quantity of Alveolar Bone Near the Target Tooth Were Reduced With the Increased Number of Micro-Osteoperforations

All groups were examined using the serial images for the quantitative analysis of the bone volume and trabecular region of the molar (Figures 1A–J). Analysis was conducted accordingly by measuring bone mineral density (BMD), bone volume fraction (BVF), bone volume (BV), trabecular thickness (Tb. Th), trabecular number (Tb. N), and trabecular separation (Tb. Sp). The bone structure and mineral contents, i.e., the BMD, BV, and BVF (Figures 1K–M) were lower in the 4MOP+OTM group compared to those of the 0MOP+OTM and Sham groups. In addition, the 4MOP+OTM group showed a significant difference in the Tb. N (Figure 1N) compared with that of the 0MOP+OTM group. The 2MOP+OTM group exhibited no differences between groups. The Tb. Th, Tb. Sp showed significant differences between the 4MOP+OTM and Sham groups (Figures 1O,P). These results suggest that the 4MOP+OTM group showed active bone and mineral reduction.

### The Number of Micro-Osteoperforations Applied Did Not Affect Root Resorption

The effect of the number of MOPs on the rate of tooth movement on the root surface was studied using micro-CT volumetric analysis by the VRR and ARR. The distal roots were covered by a thick cementum with a rough, irregular surface that occasionally contained resorption craters. These wide, shallow, and deep resorption craters were scattered primarily on the acellular area and mesial portions of the distal roots (Figure 2A rectangular). The root resorption in the MOP groups were detected (Figures 2B–I arrows), but were not significant in all MOP groups (Figures 2J,K). Based on this result, MOP operations did not significantly influence root resorption during OTM.

### The Number of Micro-Osteoperforations Applied With Orthodontic Tooth Movement Increased Bone Remodeling in the Compression Side of the Periodontium

**FIGURE 4:**
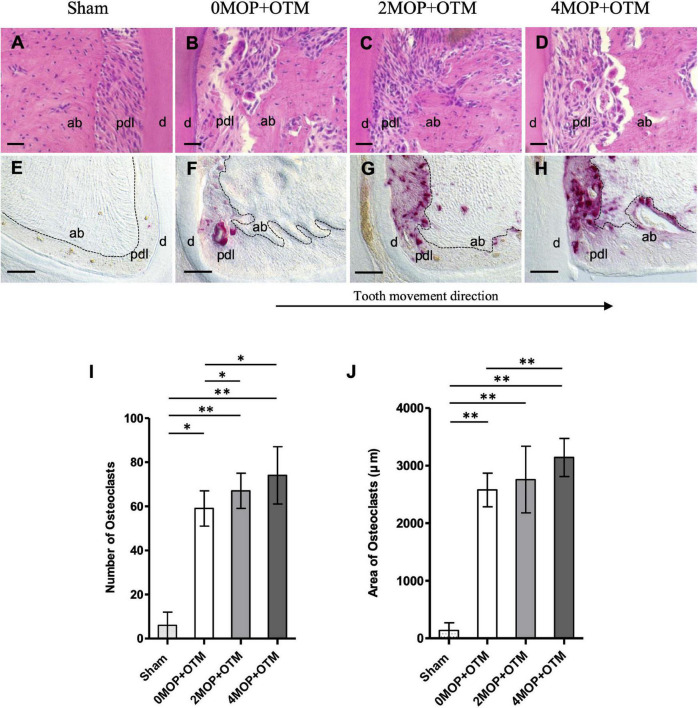
Morphological changes and osteoclast activity. **(A–D)** H&E staining show the morphological changes on the compression site of the distal root after the experimental period. **(E–H)** TRAP staining shows the comparison of the osteoclast activity among groups. **(I)** Quantification analysis of the number of osteoclast cells showed an increase in the 2MOP+OTM group (67 ± 8; *p* < 0.05) and 4MOP+OTM group (74 ± 13; *p* < 0.05) compared with the 0MOP+OTM group (59 ± 8). Sham group (6 ± 6; *p* < 0.01) was significantly lower than other groups. **(J)** The area of osteoclasts was increased in the 4MOP+OTM group (3141.76 ± 330.67 μm; *p* < 0.01) compared with the 0MOP+OTM group (2577.52 ± 291.84 μm). Further, the Sham group (136.05 ± 132.17 μm; *p* < 0.01) was significantly lower compared with other groups. (*N* = 8/each group) (d, dentin; pdl, periodontal ligament; ab, alveolar bone; dashed lines, outline of PDL area; Scale bars = 30 μm **(A–D)**; Scale bars = 50 μm **(E–H)**; data are expressed as mean ± standard deviation. Tested by Mann-Whitney *U* test, at *p < 0.05; ^**^p < 0.01).

Besides root resorption, bone resorption and osteoclast cells were analyzed on the alveolar bone surrounding the distal root. Resorption of alveolar bone on the compression side were detected and increased with the number of MOPs in the OTM group, compared to no resorption in the Sham group (Figures 4A–D). Through TRAP staining, osteoclasts were observed on the compression side of the periodontium, whereas no signal was noted on the tension side of the distal root (Figures 4E–H). The number (Figure 4I) and area (Figure 4J) of osteoclasts were greater in the 2MOP+OTM and 4MOP+OTM groups compared to the 0MOP+OTM group. Further, the Sham group exhibited a significantly lower TRAP signal compared to the other groups. In summary, following MOP with OTM, the periodontium exhibited strong bone remodeling with osteoclastic bone resorption.

### The Number of Micro-Osteoperforations Was Correlated With Increased Tooth Movement, Decreased Molar Angulations, and Reduced Bone Parameters

Correlation tests were performed on several variables related to the number of MOPs (Table 1). Analyses were conducted between experimental groups (0MOP+OTM, 2MOP+OTM and 4MOP+OTM/12 micro-CT data per group). The number of MOP was positively correlated with tooth movement distance, whereas change in molar inclination was negatively correlated with bone parameters. VRR was positively correlated with the distance of tooth movement. Moreover, bone mineral density and bone volume were negatively correlated with tooth movement. Additionally, VRR was not correlated with the number of MOPs and bone mineral density.

**TABLE 1:** Correlation analysis of number of MOPs, volume of root resorption and Bone mineral density, and Bone volume.

	Tooth movement distance		Change of molar inclination		BMD		*BV*		Volume of root resorption	
										
Parameters	Correlation coefficient	*p*-value	Correlation coefficient	*p*-value	Correlation coefficient	*p*-value	Correlation coefficient	*p*-value	Correlation coefficient	*p*-value
Number of MOPs	0.41	0.01**	–0.44	0.007*	–0.42	0.01**	–0.42	0.01*	0.23	0.17
Volume of root resorption	0.33	0.04*	–0.05	0.73	–0.03	0.82	–0.11	0.5	–	–
BMD	–0.39	0.01**	0.23	0.17	–	–	0.94	0.00***	–0.03	0.14
BV	–0.40	0.01**	0.15	0.37	0.94	0.00**	–	–	–0.11	0.5

Data are presented as Non-parametric test Spearman correlation analysis results. N = 12 per group. MOPs, micro-osteoperforations; BMD, mg/cm^3^, bone mineral density; BV, mm^3^, bone volume; Significance level was predetermined as *p < 0.05; **p < 0.01; ***p < 0.001.

### Wnt Signaling and Remineralization in Periodontium Where Micro-Osteoperforations Applied

**FIGURE 5:**
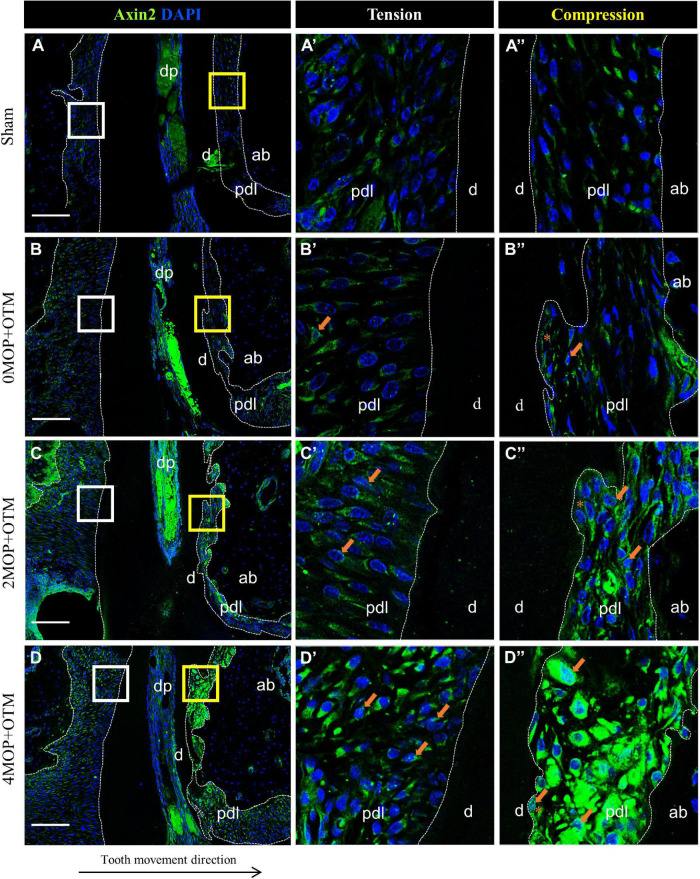
Differential expression of Axin2 dependent to the number of MOP. Wnt signaling regulator, Axin2 expression was detected in PDL area surrounding distal root. **(A)** In sham group, Axin2 was evenly expressed in both sides of PDL. **(A’)** High magnification of tension side (*white box of* **A**) and **(A”)** compression side (*yellow box of A*) PDL of the Sham group. **(B)** Axin2 expression in 0MOP+OTM group. **(B’)** High magnification of tension side (*distal*) and **(B”)** compression side (*mesial*) side of PDL. Relatively stronger expression of Axin2 (*arrow*) than the surrounding area was observed on root resorption area. **(C)** Axin2 expression in 2MOP+OTM group. **(C’)** In tension side, Axin2 expression was slightly increased than 0MOP+OTM group. **(C”)** In compression side, moderate to strong expression was observed (*arrows*). **(D)** In 4MOP+OTM group, Axin2 expression was increased compared to other groups. The expression was stronger at root resorption area of compression side. **(D’)** The tension side showed moderate to strong expression. **(D”)** The compression side, intensively strong expression of Axin2 was detected. (Dashed lines, outlines of PDL area; d, dentin; p, periodontal ligament; ab, alveolar bone; dp, dental pulp; asterisks (*) indicate root resorption area; arrow, examples of single stained cells; Scale bars = 100 μm).

**FIGURE 6:**
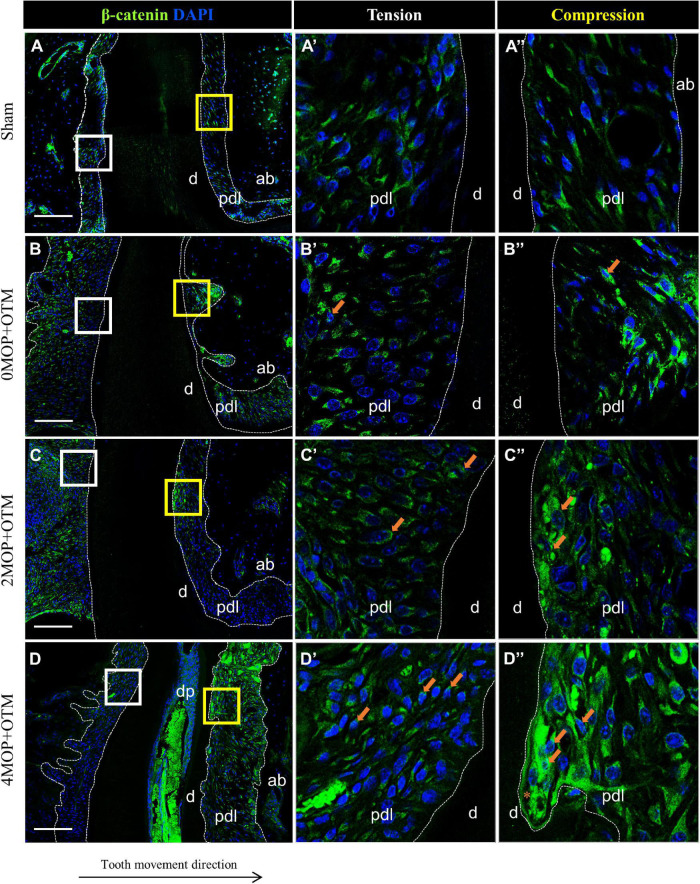
Differential expression of Wnt/β-catenin dependent to the number of MOP. **(A)** In sham group, Wnt/β-catenin was evenly expressed in both sides of PDL. **(A’)** High magnification of tension side. **(A”)** High magnification of compression side of PDL. **(B)** β-catenin expression in 0MOP+OTM group. **(B’)** High magnification of tension side and **(B”)** Compression side. Relatively stronger expression of β-catenin (*arrow*) on both side of PDL than Sham group. **(C)** 2MOP+OTM group. **(C’)** The tension side was moderate increased. **(C”)** The compression side was slightly increased expression (*arrows*) than 0MOP+OTM group. **(D)** In 4MOP+OTM group, β-catenin expression was strongly increased compared to other groups. **(D’)** In tension side showed moderate to strong expression. **(D”)** In compression side, intensively strong expression of β-catenin was detected especially on root resorption area. (Dashed lines, outlines of PDL area; d, dentin; p, periodontal ligament; ab, alveolar bone; dp, dental pulp; asterisks (*) indicate root resorption area; arrow, examples of single stained cells; Scale bars = 100 μm).

Wnt-responsive Axin2 (Figures 5A–D) was expressed on both sides of the periodontium on MOP operated groups compared with Sham (Figures 5A’,A) and 0MOP+OTM groups (Figures 5B’,B” asterisk). In addition, strong expression of Axin2 was observed at the PDL of both sides (Figures 5C’–D” arrows) and especially in crater area of the 4MOP+OTM group (Figure 5D” asterisk). Similar to Axin2, the expression of β-catenin which is a canonical Wnt pathway mediator was examined between the groups (Figures 6A–D). There was no noticeable change in both sides of the Sham group (Figures 6A’,A”) and tension sides of the experimental groups (Figures 6B’–D’ asterisk), whereas dramatic increase of β-catenin expression was detected on the compression sides of the experimental groups (Figures 6B”–D”). In addition, this increase in expression on the compression sides appeared to be correlated to the increasing number of MOPs and concentrated in the locations receiving the most orthodontic force or crater areas. These results suggest that the MOP procedure enhances the Wnt/β-catenin signaling pathway.

**FIGURE 7:**
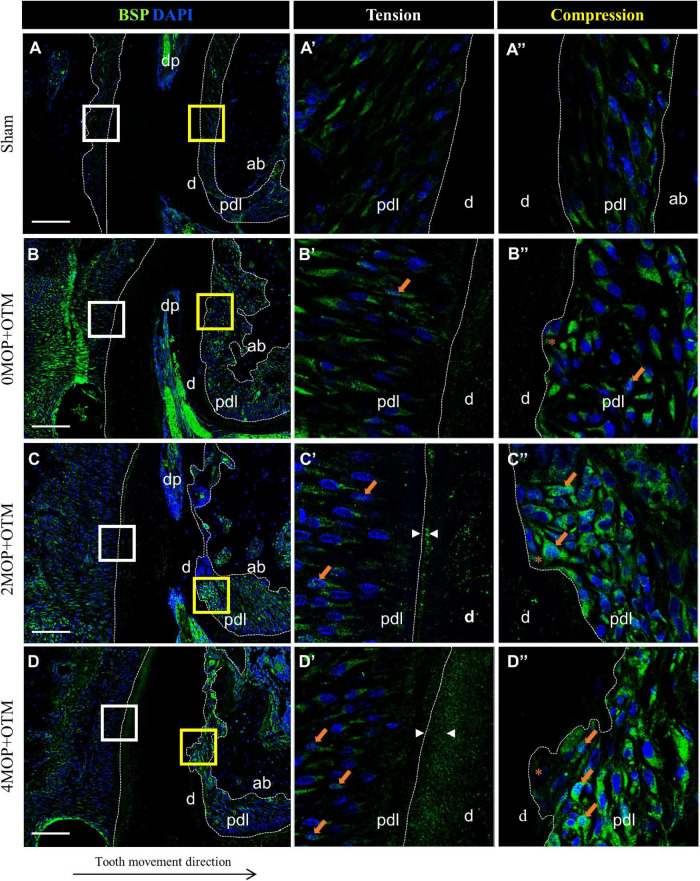
Differential expression of BSP dependent to the number of MOP. **(A)** In sham group, BSP was evenly expressed in both sides of PDL. **(A’)** High magnification of tension side. **(A”)** High magnification of compression side of PDL. **(B)** BSP expression in 0MOP+OTM group. **(B’)** High magnification of tension side and **(B”)** Compression side. BSP expression (*arrow*) was observed stronger in both side of periodontium. **(C)** BSP expression in 2MOP+OTM group. **(C’)** In tension side showed moderate to high expression (*arrows*) in PDL, and moderate mineralization (*arrowhead*) was showed on cementum layer. **(C”)** In compression side, strongest expression was detected on root resorption area compared to the 0MOP+OTM group. **(D)** In 4MOP+OTM group, BSP expression was showed highly increased cementum mineralization on tension side (*arrowhead*) and increased expression on compression side (*arrows*) compared to other groups. **(D’)** The tension side was observed slight moderate expression on PDL tissue and strongest mineralization of cementum layer. **(D”)** The compression side, slightly moderate expression of BSP was detected on crater area compared to 2MOP+OTM group. (Dashed lines, outlines of PDL area; d, dentin; p, periodontal ligament; ab, alveolar bone; dp, dental pulp; asterisks (*) indicate root resorption area; arrow, examples of single stained cells; arrowhead, mineralization thickness of acellular cementum; Scale bars = 100 μm).

**FIGURE 8:**
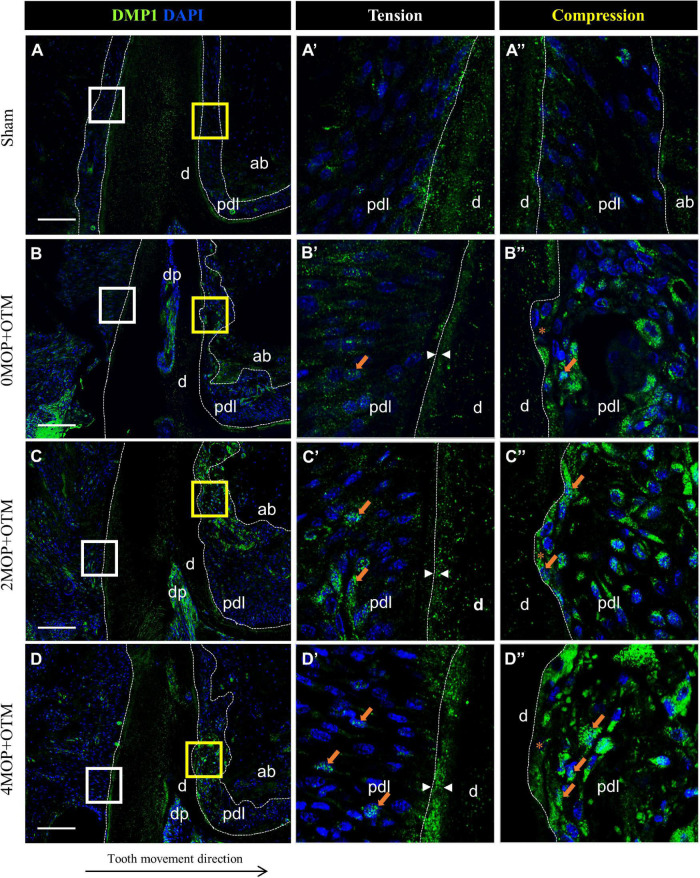
Differential expression of DMP1 dependent to the number of MOP. **(A)** In Sham group, DMP1 was evenly expressed in both sides of cementum layer and PDL tissue. **(A’)** High magnification of tension side and **(A”)** Compression side PDL of the Sham group. **(B)** DMP1 expression in 0MOP+OTM group. **(B’)** Tension side and **(B”)** Compression side. Relatively stronger expression of DMP1 (*arrow*) was observed both side of PDL tissue and similar mineralization of cementum compared to Sham group. **(C)** DMP1 expression in 2MOP+OTM group. **(C’)** In tension side, DMP1 was observed moderate to high expression on PDL and mineralized cementum mass (*arrowhead*) than 0MOP+OTM group. **(C”)** In compression side (*crater*), stronger expression (*arrows*) was detected. **(D)** In 4MOP+OTM group, DMP1 was expressed (*arrows*) in similar both side of PDL tissue and stronger cementum mineralization (*arrowhead*) was showed in 4MOP+OTM group compare with 2MOP+OTM group. **(D’)** In tension side, high expression on PDL (*crater*) and intensively strong mineralization on cementum layer was observed. **(D”)** In compression side was detected strong expression on root resorption area. (Dashed lines, outlines of PDL area; d, dentin; p, periodontal ligament; ab, alveolar bone; dp, dental pulp; asterisks (*) indicate root resorption area; arrow, examples of single stained cells; arrowhead, mineralization thickness of acellular cementum; Scale bars = 100 μm).

To investigate the mineralization effects after the MOP procedure, BSP (Figure 7) and DMP1 (Figure 8) expression was examined in the periodontium. BSP was accumulated in tension side cementum of MOP operated groups (Figures 7C’,D’ arrowheads), and wider mineralization was observed in the 4MOP+OTM group than in the 2MOP+OTM group. On the compression side, BSP expression in the MOP operated groups in PDL increased (Figures 7C”,D” arrows) compared with Sham and 0MOP+OTM groups (Figures 7A”,B”). DMP1 was expressed on both sides of the PDL and cementum. Particularly on the tension side, a difference of DMP1 was found in the cementum, and it increased in the experimental groups compared to the Sham group, with a tendency to increase according to the number of MOPs (Figures 8B’–D’ arrowheads). However, DMP1 was expressed in the PDL of the compression side (Figures 8A”–D”). The expression displayed a tendency to increase according to OTM and number of MOPs. In addition, DMP1 was absent in the root surface crater area, but strong expression was observed in PDL tissue close to the root surface cementum.

## Discussion

We demonstrated that the amount of tooth movement differed depending on the presence and number of MOPs with OTM. The 4MOP+OTM group exhibited 1.31 times greater tooth movement compared with the 0MOP+OTM group after 14 days. The faster tooth movement with MOP accelerated RAP in the target tooth. These findings are in agreement with those of other authors (Teixeira et al., 2010; Dutra et al., 2018; Sugimori et al., 2018) who found 1.35–2.13 times faster rate of tooth movement in a (2–4) MOP group compared to a control group. In addition, a report using a rat model showed that a 10-MOP group significantly increased tooth movement during the initial phase (within 7 days after MOP) (Baloul et al., 2011). However, from previous studies, it was concluded that a small number of MOPs (1–2), the magnitude of the force, presence of a flap, and age of the subjects had no impact on the rate of OTM (Ren et al., 2003; Murphy et al., 2014; Zuppardo et al., 2020). Our study also showed that there was no significant difference in 0MOP+OTM vs. 2MOP+OTM. Taking all these findings into consideration, we speculated that the number of MOPs had a major effect on the amount of tooth movement, and 4MOP+OTM could sharply induce RAP in the initial phase of OTM.

Micro-CT was used to evaluate various bone parameters such as the BMD, BV, BVF and Tb. N. The bone parameters significantly decreased in the 4MOP+OTM group compared with the 0MOP+OTM group. Our results confirmed those of previous animal studies (Chang et al., 2019; Liu et al., 2021) which showed significantly decreased alveolar bone density from alveolar decortication compared to the control group. In addition, inclination of the molar Therefore, the 4MOP+OTM group can reduce bone mineralization and may indicate a highly active bone catabolism during OTM.

The volume and area of root resorption increased significantly in the experimental groups compared to those of the Sham group, but the values were not statistically different among the experimental groups. Similarly, some studies reported that the amount of root resorption was not significantly different between various numbers of MOPs and the control group (Cheung et al., 2016; Kurohama et al., 2017). Chen et al. (2020) reported that the volume of buccal root significantly differed but volumes of other roots exhibited no difference between 2 and 4 decortications. On the other hand, enhanced alveolar bone turnover by MOP associated with an increase in osteoclast activity has been found to induce the root resorption process (Teixeira et al., 2010). From our results, we speculate that the 4MOP+OTM group might induce a considerable number of inflammatory mediators around the periodontal tissue and increase trabecular bone mineralization (van Gemert et al., 2019) after 2 weeks. However, this pattern of mineralization was on the surface of the bone and might not cause an obvious increase in root resorption.

The histological result showed that, all experimental groups exhibited a significantly increased number of active osteoclasts within the compression (mesial) side of the distal root compared to the Sham group. The results of quantification analysis after MOP agree with the results of previous studies (Tsai et al., 2016). They observed that osteoclast activity significantly increased with (3) MOP (12.9–55%) procedure. The 4MOP+OTM (25.4 %) group displayed significantly higher osteoclast activity compared with the 0MOP+OTM group. Meanwhile, the 2MOP+OTM group did not show any significant difference compared with the 4MOP+OTM group. On the contrary, another study reported that OTM with various numbers of MOPs revealed a significantly different number of osteoclasts. Different protocols with different force levels, and quantification methods of osteoclast positive cells could achieve different results (Chang et al., 2019). However, in the present study, the effect of the number of MOPs was limited to an increase in osteoclast activity during OTM.

Wnt-responsive cells displayed greater expression in some tissues by the corticotomy in a mouse experimental model (Liu et al., 2021). In our study, in MOP operated groups, Axin2 cells were highly expressed and activated in both sides of PDL tissue in the distal root than that of the other groups, and exhibited particularly strong expression in the crater area by root resorption. The previous animal study (Liu et al., 2021) using qRT-PCR reported that the level of Axin2 significantly increased with MOP by analyzing periodontal tissue surrounding roots of the molar. Considering the crater area showing high expression of Axin2, MOP operation is shown to affect regeneration of the cementum layer in the resorptive area of the root surface. However, more evidence with quantitative analysis showing that MOP accelerates Axin2 expression for the cementum and tissue regeneration in PDL area is required.

As a previous study suggested (Babb et al., 2017), the Wnt/β-catenin activated signaling pathway, may work synergistically with other damages in response to trauma and injury. In our study, 0MOP+OTM group showed similar results to that of a previous study (Fu et al., 2016), which β-catenin signaling pathway was significantly activated on the PDL of the tension site during OTM. Moreover, in our MOP operated groups, Wnt/β-catenin was activated not only great expansion on the tension site, but was also highly expressed in the crater area of the compression site. Therefore, the current study showed that the MOP procedure upregulated Wnt/β-catenin in the PDL tissue, and promoted stronger expression of the signaling compared with the 0MOP+OTM group.

BSP expression and activation in the MOP operated groups were high in the cementum and PDL tissue compared to those of the Sham group. Other studies using qRT-PCR, suggested that the BSP increased in periodontium at an early stage (7 and 14 days) of the OTM with alveolar decortications (Baloul et al., 2011; Zhou et al., 2019), which was confirmed in our present study by immunofluorescence. The present study suggests that BSP can promote strong expression in the cementum area after MOP operations and may affect mineralization of the cementum in the crater area.

Finally, DMP1 was expressed in both sides of periodontium of the root surface in 0MOP+OTM group compared with the Sham group. A previous study involving OTM reported that the mRNA level of DMP1 showed higher expression on the tension side of PDL tissue compared to the compression side of the root (Gluhak-Heinrich et al., 2003). Meanwhile, our MOP operated groups showed increases of DMP1 expression with increased numbers of MOPs in both sides of PDL tissue, particularly in the compression side. However, DMP1 expression was absent in the crater area (compression) of the root cementum in MOP operated groups. Meanwhile, DMP1 was widely expressed in the tension side of the cementum layer compared with the 0MOP+OTM and Sham groups. From these results we speculate that, MOP operations increase the expression of DMP1, which may be related to cementum mineralization on the tension side and root resorption repair on the compression side.

In summary, the RAP from MOP increased the rate of tooth movement through reduction of bone volume and bone density in the operation site. However, the number of MOP did not increase root resorption significantly, although TRAP activity was increased in the compression site of PDL and tension site of the cementum. The increased number of MOP accompany a slight increase in the expression of regenerative marker, which suggests a homeostatic regulation concept for bone remodeling.

This study had a few limitations. We only measured tooth movement for a short period of time. In addition, there are morphological and physiological differences between rat and human alveolar bone. In rats, the quality and quantity are different; therefore, bone remodeling is also different. The alveolar bone of rodents is denser and exhibits no osteoid remodeling (secondary remodeling). Humans have more osteoid tissue along the alveolar bone surface (Reitan and Kvam, 1971). Therefore, a different animal model is required to simulate human alveolar bone more closely.

Despite these limitations, this *in-vivo* study aids our understanding of the effect of the number of MOPs on the surrounding alveolar bone. As mentioned previously, MOP is one of the surgical interventions to accelerate tooth movement. However, the effect of the number of MOPs and the side effects of root resorption on tooth movement were not clearly understood. This study was designed to monitor and evaluate these effects. We attempted to define the maximum and minimum number of perforations (2 and 4) for the acceleration of tooth movement. Our animal study revealed the optimal amount of damage incurred in the alveolar bone to ensure efficient tooth movement without causing significant damage to the root surface. In addition, tissue biomarkers proliferated and there was a rapid repair process after MOP operations. However, there is still concern about the clinical application of the present results because humans and mice differ regarding bone remodeling and tissue regeneration.

Our future studies will focus on understanding the signaling pathways associated with the MOP group using *in-vitro* gene expression of osteoclasts and cementoblasts in the periodontium, while considering the longer term effects of MOP. Further studies are required to assess the optimal frequency of MOP with minimum side effects.

## Conclusion

Our results support that the MOP procedure was effective in accelerating tooth movement by decreasing bone density as evidenced by an increased number of osteoclasts; the number of MOPs did not directly correlate with root resorption. Therefore, it is suggested that the minimally invasive procedure may be effective in tooth movement under cementum regeneration. This study established a mouse model for validating tooth movement and the side effects in the periodontium with the MOP procedure.

## Data Availability Statement

The original contributions presented in the study are included in the article/Supplementary Material, further inquiries can be directed to the corresponding author/s.

## Ethics Statement

The animal study was reviewed and approved by the Institutional Animal Care and Use Committee, Yonsei Medical Centre, Seoul, Korea (Approval No. 2018-0052).

## Author Contributions

J-YC and H-SJ designed study and performed the research. TE, D-JL, E-JK, E-HC, and C-JH contributed to analysis tool. S-JK and JL discussed the data. J-YC, TE, D-JL, JL, and E-HC performed the experiment. TE and D-JL analyzed the data. J-YC, TE, and D-JL wrote the manuscript. YC, TE, D-JL, and H-SJ revised manuscript. All authors contributed to the article and approved the submitted version.

## Conflict of Interest

The authors declare that the research was conducted in the absence of any commercial or financial relationships that could be construed as a potential conflict of interest.

## Publisher’s Note

All claims expressed in this article are solely those of the authors and do not necessarily represent those of their affiliated organizations, or those of the publisher, the editors and the reviewers. Any product that may be evaluated in this article, or claim that may be made by its manufacturer, is not guaranteed or endorsed by the publisher.
