# Supplementary figures and images for: Effect of the Number of Micro-Osteoperforations on the Rate of Tooth Movement and Periodontal Response in Mice
Source: Front Physiol. 2022 Mar 3;13:837094. doi: 10.3389/fphys.2022.837094 (PMC8928525; doi:10.3389/fphys.2022.837094)

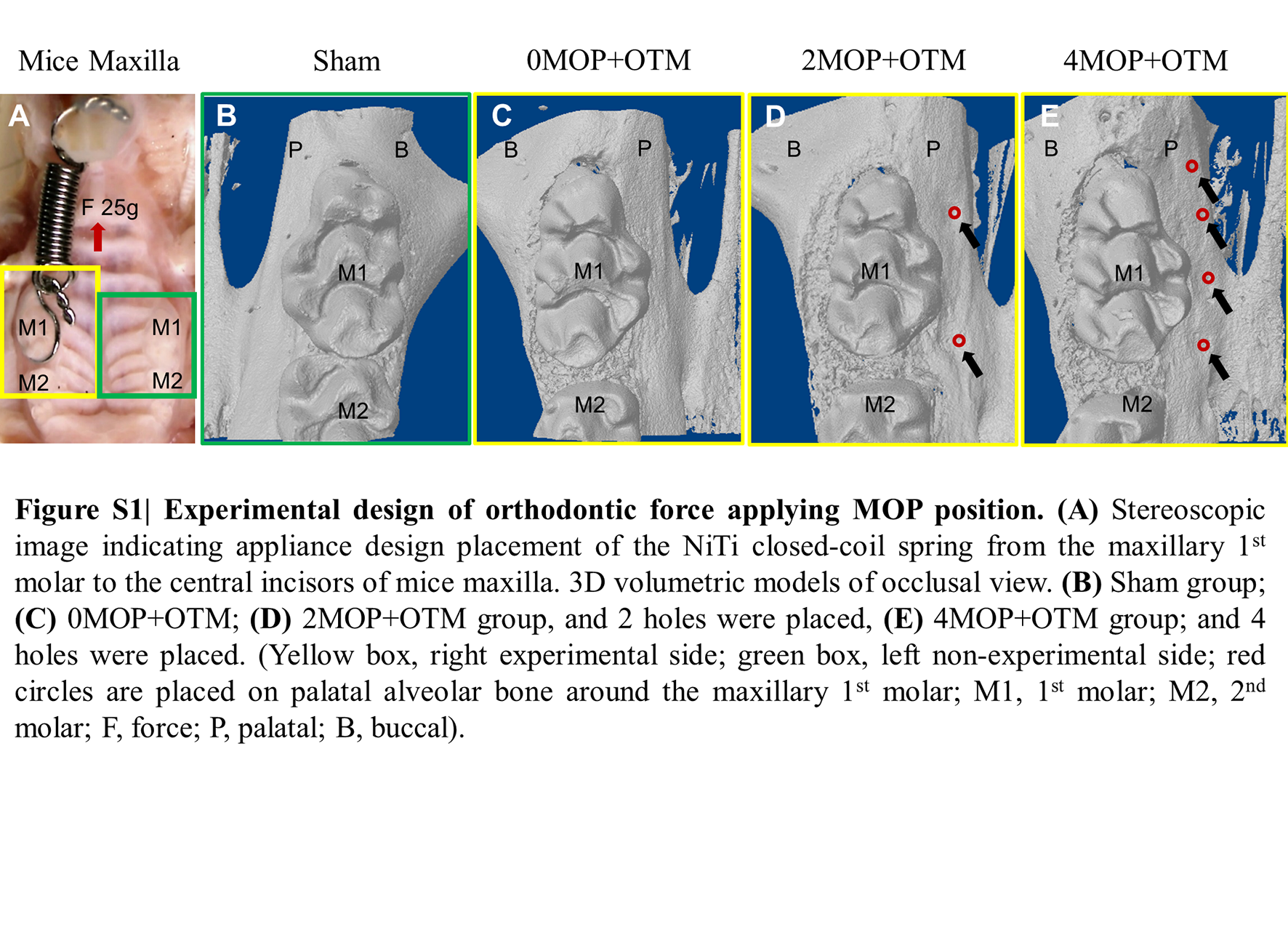

Supplement: Supplementary file 1 [file Image_1.TIF]

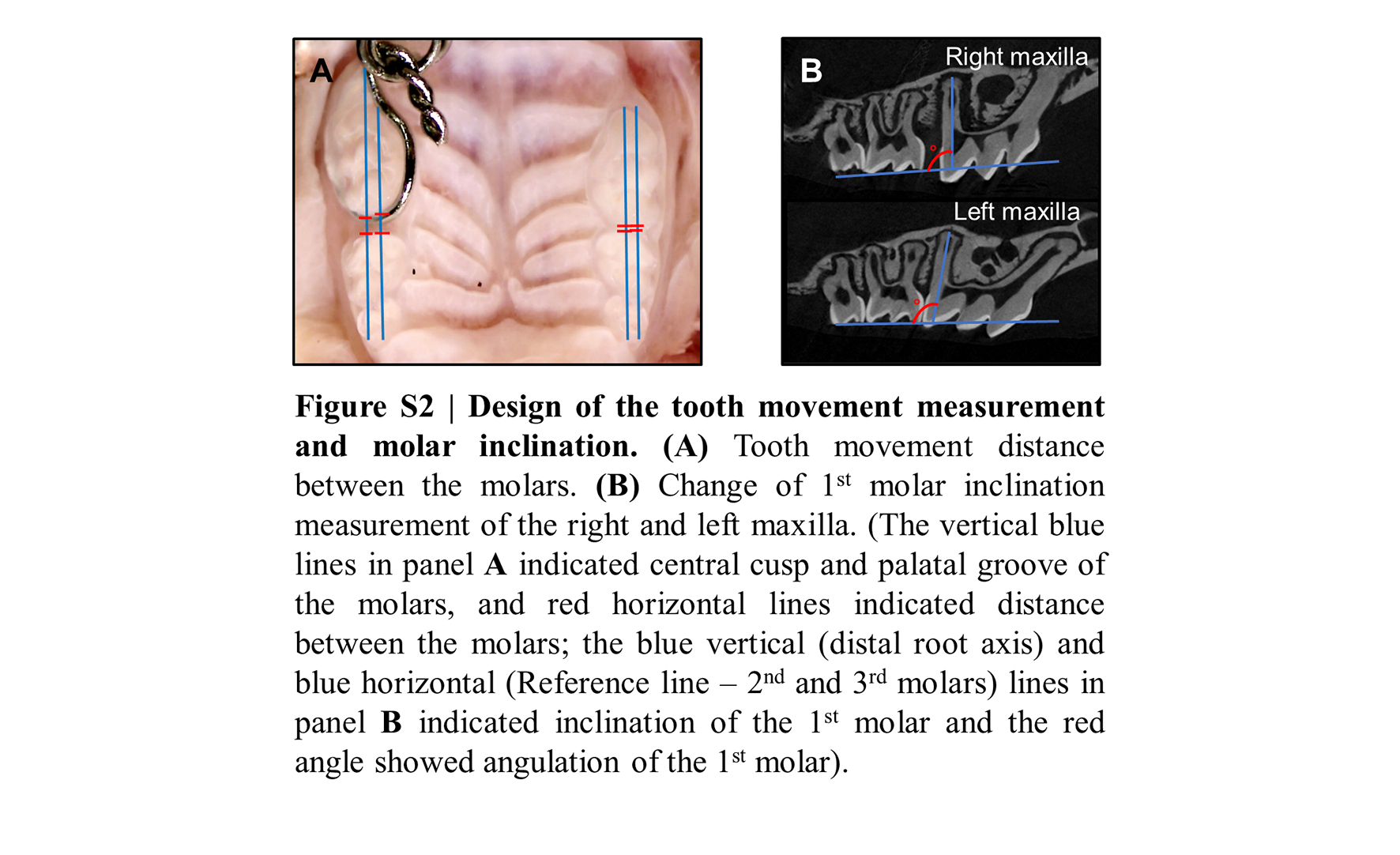

Supplement: Supplementary file 2 [file Image_2.TIF]

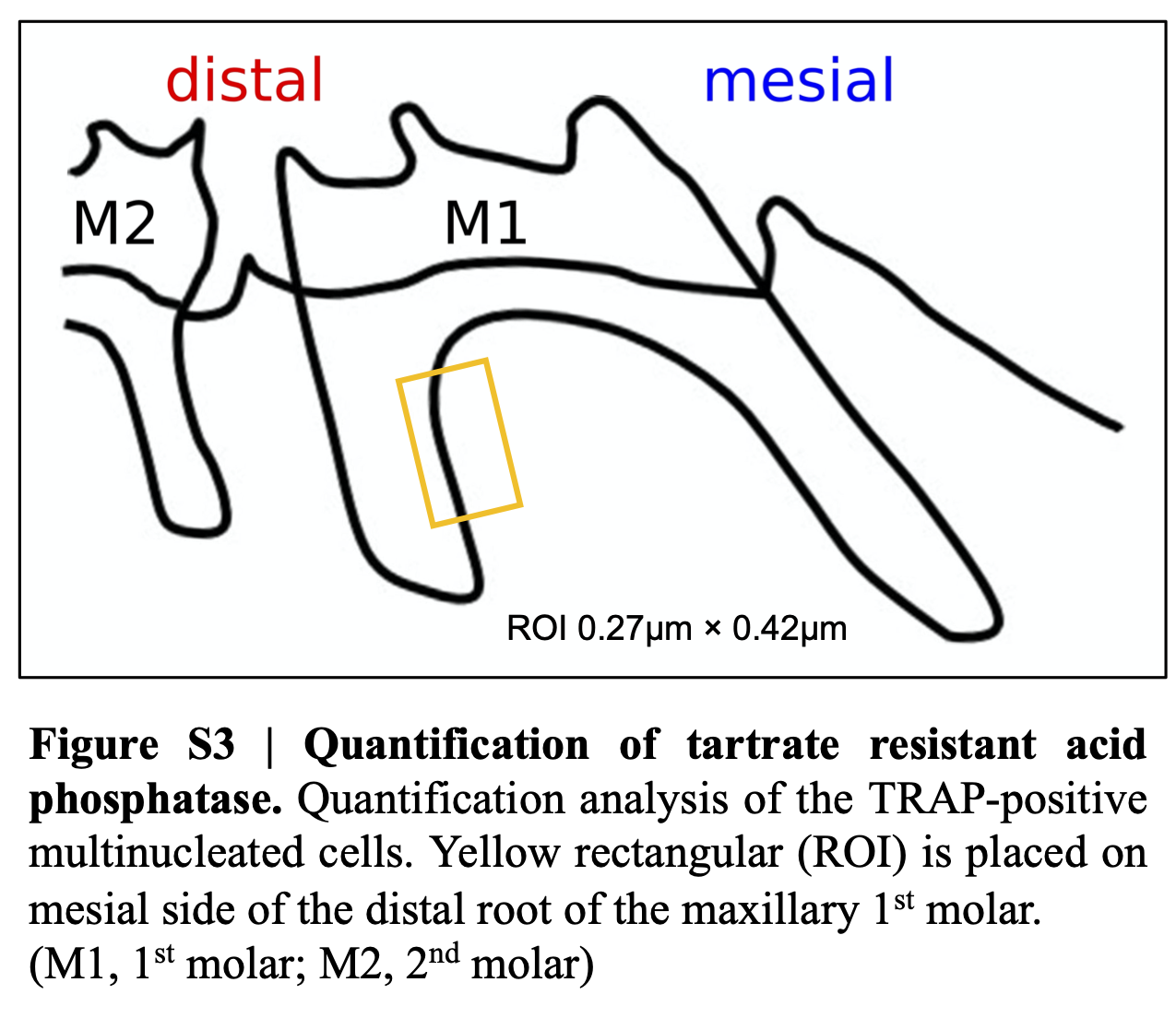

Supplement: Supplementary file 3 [file Image_3.TIFF]
